# Supplementary material for: A UMLS-based spell checker for natural language processing in vaccine safety
Source: BMC Med Inform Decis Mak. 2007 Feb 12;7:3. doi: 10.1186/1472-6947-7-3 (PMC1805499; doi:10.1186/1472-6947-7-3)
Supplement: Additional file 13 — RAP application directory. Sets up directory for RAP (RDF application for PHP) source code [file 1472-6947-7-3-S13.gz › rap/doc/phpdoc/default/RDFS.html]

xml version="1.0" encoding="iso-8859-1"?


Docs For Class RDFS


## Class RDFS

Description

Description |
Methods (details)

Located in /vocabulary/RDFS\_C.php (line 22)

Method Summary

Description |
Methods (details)

void
COMMENT
()

void
CONTAINER
()

void
CONTAINER\_MEMBERSHIP\_PROPERTY
()

void
DATATYPE
()

void
DOMAIN
()

void
IS\_DEFINED\_BY
()

void
LABEL
()

void
LITERAL
()

void
MEMBER
()

void
RANGE
()

void
RDFS\_CLASS
()

void
RESOURCE
()

void
SEEALSO
()

void
SUB\_CLASS\_OF
()

void
SUB\_PROPERTY\_OF
()

Methods

Description |
Methods (details)

COMMENT (line 90)

void

COMMENT
()

CONTAINER (line 48)

void

CONTAINER
()

CONTAINER\_MEMBERSHIP\_PROPERTY (line 54)

void

CONTAINER\_MEMBERSHIP\_PROPERTY
()

DATATYPE (line 42)

void

DATATYPE
()

DOMAIN (line 72)

void

DOMAIN
()

IS\_DEFINED\_BY (line 108)

void

IS\_DEFINED\_BY
()

LABEL (line 84)

void

LABEL
()

LITERAL (line 30)

void

LITERAL
()

MEMBER (line 96)

void

MEMBER
()

RANGE (line 78)

void

RANGE
()

RDFS\_CLASS (line 36)

void

RDFS\_CLASS
()

RESOURCE (line 24)

void

RESOURCE
()

SEEALSO (line 102)

void

SEEALSO
()

SUB\_CLASS\_OF (line 60)

void

SUB\_CLASS\_OF
()

SUB\_PROPERTY\_OF (line 66)

void

SUB\_PROPERTY\_OF
()

Documentation generated on Fri, 17 Dec 2004 16:17:16 +0100 by phpDocumentor 1.3.0RC3
